# Argonaute 2 is a key regulator of maternal mRNA degradation in mouse early embryos

**DOI:** 10.1038/s41420-020-00368-x

**Published:** 2020-11-27

**Authors:** Jia-Ming Zhang, Wei-Bo Hou, Jia-Wei Du, Ming Zong, Kai-Lun Zheng, Wei-Jia Wang, Jia-Qiang Wang, Heng Zhang, Yan-Shuang Mu, Zhi Yin, Chun-Ming Ding, Qing-Yuan Sun, Zhong-Hua Liu, Qing-Ran Kong

**Affiliations:** 1 Key Laboratory of Animal Cellular and Genetics Engineering of Heilongjiang Province, College of Life Science, Northeast Agricultural University, Harbin, 150030 Heilongjiang China; 2 School of Laboratory Medicine and Life Sciences, Wenzhou Medical University, Wenzhou, Zhejiang Province China; 3 Key Laboratory of Laboratory Medicine, Ministry of Education, Wenzhou Medical University, Wenzhou, Zhejiang Province China; 4 Fertility Preservation Lab, Reproductive Medicine Center, Guangdong Second Provincial General Hospital, Guangzhou, 510317 China

**Keywords:** Cell lineage, Gene regulation

## Abstract

In mammalian early embryos, the transition from maternal to embryonic control of gene expression requires timely degradation of a subset of maternal mRNAs (MRD). Recently, zygotic genome activation (ZGA)-dependent MRD has been characterized in mouse 2-cell embryo. However, in early embryos, the dynamics of MRD is still poorly understood, and the maternal factor-mediated MRD before and along with ZGA has not been investigated. Argonaute 2 (*Ago2*) is highly expressed in mouse oocyte and early embryos. In this study, we showed that *Ago2*-dependent degradation involving RNA interference (RNAi) and RNA activation (RNAa) pathways contributes to the decay of over half of the maternal mRNAs in mouse early embryos. We demonstrated that AGO2 guided by endogenous small interfering RNAs (endosiRNAs), generated from double-stranded RNAs (dsRNAs) formed by maternal mRNAs with their complementary long noncoding RNAs (CMR-lncRNAs), could target maternal mRNAs and cooperate with P-bodies to promote MRD. In addition, we also showed that AGO2 may interact with small activating RNAs (saRNAs) to activate *Yap1* and *Tead4*, triggering ZGA-dependent MRD. Thus, *Ago2*-dependent degradation is required for timely elimination of subgroups of maternal mRNAs and facilitates the transition between developmental states.

## Introduction

Growing oocytes possess high transcriptional activity and accumulate large amounts of maternal mRNAs and proteins, which control the initial stages of development^1,2^. During maternal-to-zygotic transition (MZT), the majority of maternal mRNAs are eliminated, and the zygotic genome becomes transcriptionally active^3,4^. MRD is required to remove repressive factors and enable ZGA^5–7^.

MRD occurs during oogenesis, beyond fertilization, and is accomplished along ZGA. It acts during two sequential processes from oocyte to early embryo^8^. In oocyte, the maternally encoded products are exclusively used to activate the degradation pathway. For example, the RNA-binding proteins, SMAUG and BTG4, can recognize some specific maternal mRNAs and trigger MRD^9–14^. In *Drosophila*, *Zebrafish*, and *Xenopus* early embryos, small RNA, especially microRNA, has been reported to promote the degradation of their target mRNAs^15^. Recently, in mouse embryos, the ZGA-dependent maternal mRNA clearance has been characterized at 2-cell stage, and YAP1- and TEAD4-mediated zygotic transcription is crucial for the pathway^16^. However, a group of maternal mRNAs are observed to degrade rapidly in mouse 1-cell embryo. Thus, the dynamics of MRD in early embryos is still poorly understood. Before ZGA, embryogenesis is supported by maternal factors, which participate in the removal of maternal detritus and the robust activation of the embryonic genome^17–19^, suggesting the existence and functional importance of the maternal factor-mediated MRD before and along with ZGA in early embryos, but it has not been investigated.

Since the discovery of the first Argonaute gene in *Arabidopsis*, the members of this family have rapidly emerged as key components of the gene regulatory pathway at the transcriptional and post-transcriptional levels^20–22^. In mice, four AGO proteins (AGO1-4), which share structural similarities and have overlapping functions in RNAi, exist, but only AGO2 is highly expressed in oocytes and early embryos and possesses endonuclease catalytic activity^20,23^. In addition, recent reports have demonstrated that AGO2 could cooperate with saRNA to activate gene expression at the transcriptional level, implying its potency to detonate ZGA^24–27^. Moreover, *Ago2* mutant oocytes fail to progress through the first cell division event, and zygotic *Ago2* deletion leads to embryonic developmental arrest after post-implantation, while *Ago1*, *Ago3* and *Ago4* deletions are viable^23,28–31^. The features of *Ago2* indicate its potential role in early embryos. To this end, we knocked down *Ago2* (*Ago2* kd) by injection of small interfering RNA (siRNA) targeting *Ago2* and AGO2 antibodies into mouse zygotes, and demonstrated that deletion of *Ago2* impairs normal early embryonic development, accompanied by abnormal MRD and ZGA.

## Materials and methods

### Mouse experiments

All experiments were performed in accordance with the ARRIVE (Animal Research: Reporting of In Vivo Experiments) guidelines and regulations. Animal experiments were performed with 7-week-old ICR mice. Animals were maintained under a 12 h light/dark cycle and provided with food and water ad libitum in individually ventilated units.

### Embryo collection

Embryos were collected from 7-week-old F1 superovulated female mice treated with 6.5 IU of pregnant mares’ serum gonadotropin (PMSG) and, 47 h later, with 5 IU of human chorionic gonadotropin (hCG) and crossed with F1 males. Embryos were isolated in M2 medium (Sigma) and cultured in KSOM medium at 37 °C in 5% CO_2_ and fixed at the following times post-hCG injection: 20 h for the zygote, 40 h for the middle 2-cell embryo, 55 h for the early 4-cell embryo, 64 h for the 4-cell embryo, 70 h for the 8-cell embryo, 88 h for the morula and 99 h for the blastocyst. Additionally, oocytes were collected from 7-week-old ICR superovulated females at 16 h post-hCG.

### Microinjection

All small interfering RNAs (siRNAs) were purchased from GenePharma. *Ago2*-siRNAs was designed to specifically target *Ago2* and on the basis of 30%-52% GC content and avoiding of internal repeats (5′–3′). *Ago2*-siRNA1: GCAAAGAUCGCAUCUUUAATT; *Ago2*-siRNA2: GCCAGUGAUCGAGUUUGUUTT; *Ago2*-siRNA3: GCAGAAACACACCUACCUUTT; the scrambled siRNA used as negative control. All siRNAs were modified with 2′Fluoro rU/C to increase their annealing temperature. The prediction of off-target effects was described in result. To perform microinjection, zygote-stage embryos were placed in 150 µg/mL hyaluronidase (Sigma) to digest the outer granule cells. SiRNAs was centrifuged at 12,000 rpm for 10 minutes at 4 °C and placed at 4 °C for use. Then, siRNA microinjection was carried out with an Eppendorf FemtoJet microinjector and Narishige NT-88NE micromanipulators. For injection, a glass capillary Femtotip II (Eppendorf) was loaded with 2 μL of 10 µM siRNA by a microloader (Eppendorf), and the solution was injected into the cytoplasm in a 100 μL drop of M2 medium (Sigma) plus 5 μg/mL cytochalasin B (Sigma). The injection volume was approximately 2–5 pL. The injection conditions consisted of 250 hPa injection pressure, 60 hPa compensation pressure and 0.7 s injection time. Immediately after microinjection, embryos were cultured in KSOM medium at 37 °C in 5% CO_2_.

### Antibody purification

The anti-AGO2 antibody used was rabbit anti-AGO2 (Abcam, ab32381), and purchased in azide-free format and concentrated using Amicon Ultra-0.5 100 kDa centrifugal filter devices (Millipore) to remove traces of azide and replace the buffer with PBS. Prior to microinjection, antibodies were diluted in 1× PBS containing *Ago2*-siRNA (100 µM) to the following concentrations: anti-AGO2 (0.5 mg/ml) and *Ago2*-siRNA (10 µM). Prior to microinjection, antibodies-siRNA mixture was incubated on ice for 30 min. And, microinjection was performed using a micromanipulator and Eppendorf Femtojet system mounted on the OLYPUS microscope. Once the glass needle was transiently inserted into the cytoplasm, the needle was quickly withdrawn when slight swelling in cytoplasm appears, indicating the successful injection.

### Overexpression and inhibition of endosiRNA

The endosiRNAs associated with maternal mRNAs of *Zfp277*, *Brip1* and *Spin1* were verified. The sequences of the endosiRNAs are as follows (5′–3′): Zsi-1: (ACATGGTGGAGCATGTGTCCT); Zsi-2: (ACCGCCAGACTGATTTCCA); Zsi-3: (ACCAACAATGGAGGAGTGT); Zsi-4: (ACCTGAATTTTTGATCTTA); Zsi-5: (ACATTTTTTCAGGTGCTTCTC); Bsi-1: (ACAGCAATGTGGAAATGTAAGC); Bsi-2: (ACATCCCTCCATGACCTCTG); Bsi-3: (ACATCCCTCCATGACCTCTGA); Bsi-4: (ACAGTCCTGACTTCCTTTGGTGAT); Ssi-1: (ACATGTGGTTGCTGGGATTTG); Ssi-2: (ACCTATGAGAAAGACCCTGTCT); Ssi-3: (ACATCACCTATGAGAAAGACC); Ssi-4: (ACCCCTTCATGCCTTCAAA); Ssi-5: (ACCCCTTCATGCCTTCAAAA). The mimics used are small, chemically modified dsRNAs, and the sequences of one of the strands are the same with the endosiRNAs and enable upregulation of its activity. The inhibitors are small, chemically modified single-stranded RNA molecules with complementary sequences of endosiRNAs designed to specifically bind to and inhibit endosiRNA molecules and enable downregulation of endosiRNA activity.

### Immunofluorescence staining

After removal of the zona pellucida with acidic operating fluid, mouse embryos were fixed in 4% PFA for 40 minutes at room temperature (RT), followed by permeabilization in 1% Triton X-100 for 20 minutes at RT. Embryos were then blocked in blocking solution (1% BSA in PBS) for 1 h at RT after 3 washes for 5 minutes each in washing solution (0.1% Tween-20, 0.01% Triton X-100 in PBS). Incubations were performed overnight at 4 °C or for 1 h at 37 °C using the following antibodies and dilutions in blocking solution: AGO2 (1:200) and DCP1A (1:100). The next day, the embryos were washed 3 times in washing solution and incubated with secondary antibodies (goat anti-mouse IgG Alexa Fluor 647 conjugated, 1:200, Invitrogen, A32728; and donkey anti-rabbit IgG Alexa Fluor 546 conjugated, 1:500, Invitrogen, A10040) for 1 h at RT. After 5 minutes of staining with Hoechst, the embryos were washed 4 times in washing solution. Imaging of embryos in microdroplets was performed using an inverted confocal microscope. We jointly used ImageJ software and PS CC to count protein particles.

### RNA-FISH

After removal of the zona pellucida with acidic operating fluid, mouse embryos were washed twice in PBS. Then, embryos were fixed in 4% paraformaldehyde (PFA) for 40 minutes followed by 2 washes in PBS. Embryos were gradient dehydrated in ethanol and washed 2 times in PBT (1% Tween-20, in PBS). After permeabilization in permeabilizing solution (0.5% Triton X-100 in PBS), embryos were aspirated repeatedly in proteinase K (5 µg/mL) for one minute and were washed 3 times in PBT. Embryos were then hybridized in hybridization solution (50% formamide (Sigma, F9037), 2× SSC, 10% dextran sulfate (Sigma, 30915), 10 mM VRC (Sigma, 94742), 2 mg/mL BSA) containing 20 ug of DIG-labeled *lnc521* probes at 37 °C overnight (14-15 h). Embryos were washed four times in washing solution after 2 washes for 5 min each in hybridization washing solution (50% formamide, 2 × SSC) at 60 °C. We blocked the embryos in blocking solution (10% sheep serum, 0.05% BSA, in 1× PBS) for 1 h at RT followed by incubation in secondary antibody solution (Abcam, ab119349). After 4 washes for 5 min each in PBT, the embryos were stained with Hoechst for 5 min. Then, the embryos were mounted on glass slides after three washes.

### RNA extraction, reverse transcription and q-PCR analysis

Total RNA was extracted using the PureLink RNA Mini Kit (Ambion) according to the manufacturer’s instructions, and reverse transcription was performed to generate cDNA using the High Capacity cDNA Reverse Transcription Kit (Applied Biosystems). Small noncoding RNA (sncRNA) was extracted using the mirVana^TM^ microRNA Isolation Kit (Ambion) according to the manufacturer’s instructions, and reverse transcription was performed using TransScript microRNA First-Strand cDNA Synthesis SuperMix (TransGen Biotech) according to the manufacturer’s instructions. RNase-Free DNase Set (QIAGEN) was used to ensure that there was no DNA contamination. Q-PCR was performed using TB Green™ Premix Ex Taq (TaKaRa) and a 7500 q-PCR System (Applied Biosystems). The reaction parameters were as follows: 95 °C for 30 s followed by 40 two-step cycles of 95 °C for 5 s and 60 °C for 34 s. 18s rRNA and 5s rRNA were used as reference genes. Ct values were calculated using Sequence Detection System software (Applied Biosystems), and the amount of target sequence normalized to the reference sequence was calculated as 2^−^^△△Ct^. Subcellular localization analysis of *lnc521* was performed as our previous description^32^.

### RNA-seq analysis

For RNA-seq analysis of early stage embryos, FastQC was performed for Illumina reads. In addition, we employed Trim Galore software to discard low-quality reads, trim adaptor sequences, and eliminate poor-quality bases. Then, we downloaded the mouse reference genome (Genome assembly: GRCm38.p6) from Ensembl and selected HISAT2 software for read alignment. The gene-level quantification approach is to aggregate raw counts of mapped reads using HTSeq-count (parameter: “-m union”). Then, the expression level of each gene was quantified with normalized FPKM (fragments per kilobase of exon per million mapped fragments) by StringTie. Next, we used the R package DESeq2 for differential gene expression analysis. The DAVID database (https://david.ncifcrf.gov/) is an essential foundation for the success of any high-throughput gene function analysis. GO annotations were performed using the DAVID online tool on the screened differentially expressed genes.

### Maternal mRNA clustering

Maternal mRNAs with FPKM > 2 at the MII oocyte stage were retained for further analysis. The expression level of each gene was transformed by log2 (FPKM + 1) in the following analysis. Clusters I-III consist of the genes that satisfy the following criteria: cluster I: expression (MII) > expression (2-cell) + 1, expression (2-cell) < expression (4-cell) + 1, expression (2-cell) > expression (4-cell) − 1; cluster II: expression (MII) > expression (2-cell) + 1, expression (MII) < expression (2-cell) − 1, expression (2-cell) > expression (4-cell) + 1; cluster III: expression (MII) > expression (2-cell) + 1, expression (2-cell) > expression (4-cell) + 1.

### Identification of endosiRNA and microRNA target maternal mRNAs

Small RNAs were classified as previously reported. EndosiRNAs expressed in MII oocytes, 2-cell embryos and 4-cell embryos were aligned to AGO2-dependent maternal mRNAs by Bowtie (parameter: “-a -m 20 -v 2”), and endosiRNA-targeting maternal mRNAs were predicted. Additionally, microRNAs expressed in MII oocytes, 2-cell embryos and 4-cell embryos were used to align to AGO2-dependent maternal mRNAs by miRDB (http://mirdb.org/miRDB/), and a score >97 was predicted for microRNA-targeting maternal mRNAs.

### Prediction of CMR-lncRNAs and saRNAs

LncRNAs expressed in MII oocytes, 2-cell embryos and 4-cell embryos were predicted based on coding potential using PLEK^33^, CPC2^34^ and CPAT^35^ software. LncRNA expressed at the zygote, 2-cell or 4-cell stage was used to predict CMR-lncRNA. The filtered lncRNAs were aligned to endosiRNA-targeting AGO2-dependent maternal mRNAs by BWA, and lncRNAs with an alignment ratio > 90% (based on complementary sequencing) were predicted to be CMR-lncRNAs. CMR-lncRNAs were annotated by bedtools to identify the corresponding genome region. To predict saRNAs, we used small RNA-seq data (GSE83581), and the expression level was calculated and normalized using the RPM (reads per million) value. Small RNAs with lengths from 18 to 30 nt after adapter trimming and expression levels >1 at the zygote or 2-cell stage were used for further analysis. ZGA-genes were identified as described in a previous report. Then, we aligned the filtered fragments to upstream sequences within 1.0 kb of AGO2-related ZGA-gene TSSs by Bowtie to predict saRNAs and the corresponding ZGA-genes.

### Statistical analysis

Statistical analysis was performed using SPSS 13.0 for Microsoft Windows. Data are shown as the mean ± s.e.m. Most experiments included at least three independent samples and were repeated at least three times. Differences in the results of two groups were evaluated using either two-tailed Student’s *t*-test or one-way ANOVA followed by Dunnett’s test. *p < 0.05, **p < 0.01 and ***p < 0.001.

## Results

### Characteristics and degradation of maternal mRNAs

**Fig. 1:**
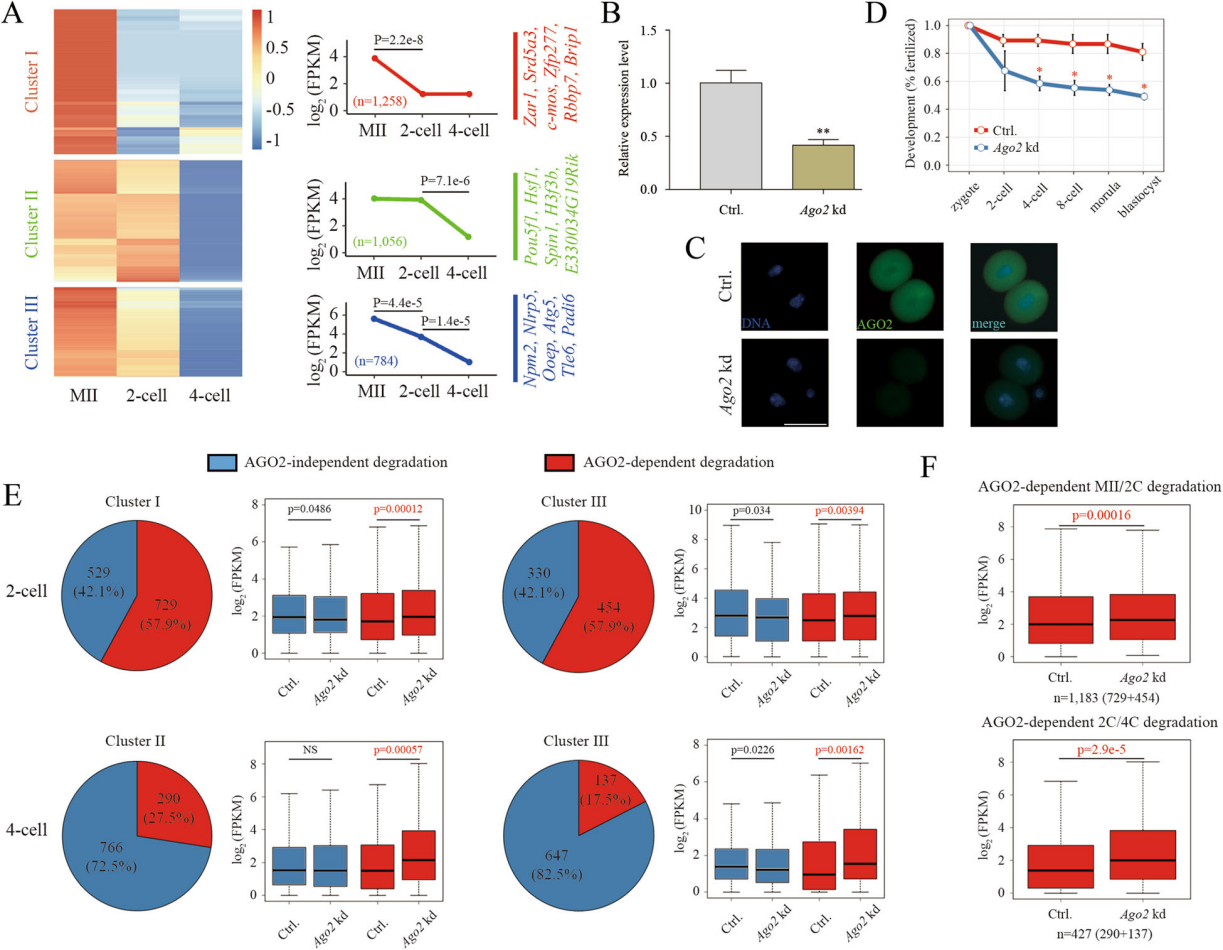
Role of *Ago2* in MRD in mouse early embryos. **A** Heat map showing the expression patterns of maternal mRNAs in MII oocytes, middle 2-cell embryos and early 4-cell embryos. We classified the maternal mRNAs into three categories according to their degradation dynamics: MII/2 C degradation, 2C/4C degradation, and continuous degradation. The expression patterns of the three clusters and representative genes are shown on the right. **B**,**C** Q-PCR and immunofluorescence analysis of 2-cell embryos injected with either *Ago2* siRNA or AGO2 antibodies, validating the *Ago2* kd efficiency. Fluorescence staining was imaged using a basal fluorescence microscope. Ctrl: control group; *Ago2* kd: *Ago2*-knockdown group. **p < 0.01, Error bars indicate the s.e.m. Scale bar, 50 µm. **D** Depletion of *Ago2* impaired the development of pre-implantation embryos in mice. The *Ago2* siRNA or AGO2 antibodies was injected into zygotes. The development rate of *Ago2* kd embryo was significantly lower than the control group at 4-cell stage. Data are presented as the mean ± s.e.m. **E** Pie chart shows the fraction of AGO2-dependent degradation in 2- and 4-cell embryos, Box plots of AGO2-independent and AGO2-dependent gene expression are shown on the right. **F** Box plots of AGO2-dependent MII/2C and 2C/4C degradation in 2- and 4-cell embryos, respectively. *Ago2*-dependent MII/2C and 2C/4C mRNA remains stable and upregulated upon *Ago2* kd.

In mice, the burst of ZGA happens during the middle-to-late 2-cell stage, and MRD occurs accompanied by ZGA and is accomplished before the 4-cell stage. In this study, to accurately identify the patterns of MRD in mouse early embryos, we take ZGA as a timing, a set of MRD occurs before ZGA, and the second set of MRD follow ZGA. On the basis of the classification rule, we performed RNA-seq on MII oocytes, middle 2-cell embryos and early 4-cell embryos. Transcripts with high expression in MII oocytes (FPKM > 2) were defined as maternal mRNAs, including some identified mRNAs, such as *Zar1*, *Hsf1*, *Ooep*, *Nlrp5* and *Npm2*, and the expression of most of these mRNAs (*n* = 3,098) decreased significantly during the transition from MII oocytes to early 4-cell embryos. The maternal mRNAs degraded during the process were categorized into three clusters. A total of 1,258 maternal mRNAs in cluster I were significantly downregulated at the 2-cell stage and remained stable during the transition from middle 2-cell to early 4-cell embryos. In contrast, cluster II, including 1,056 maternal mRNAs, showed no significant changes during the transition from MII oocytes to 2-cell embryos, but was dramatically downregulated at the 4-cell stage. Moreover, 784 maternal mRNAs that were continuously degraded during the transition from MII oocytes to early 4-cell embryos were classified into cluster III (Fig. 1A; Supplementary Table S1). Therefore, we suggest that the maternal mRNAs in cluster I could be degraded during the transition from MII oocyte to middle 2-cell embryo (MII/2 C degradation) before ZGA, while the maternal mRNAs in cluster II could be degraded during the transition from middle 2-cell to early 4-cell embryos (2C/4C degradation), which may be associated with ZGA, and the degradation of the maternal mRNAs in cluster III, termed continuous degradation, might be associated with both processes.

### *Ago2* is involved in MRD in mouse early embryos

To test the role of *Ago2* in MRD, we first examined the expression of AGO2 protein by immunofluorescence during oocyte maturation and pre-implantation embryonic development. As shown in the previous reports^23^, the signals were high in oocytes and early embryos, and peaked at blastocyst stage (Supplementary Fig. S1A), and the expression pattern was also confirmed by quantitative mass spectrometry in mouse embryos (Supplementary Fig. S1B)^36^. Then, we knocked down *Ago2* at both the RNA and protein levels by injecting siRNA targeting *Ago2* and AGO2 antibodies into zygotes. We first screened the off-targets of *Ago2*-siRNA1-3 in RefSeq database by Blastn, and three genes were found (mismatch value ≤ 2). However, except *Ago2*, *9230019H11Rik* and *Zdhhc15* were seldomly not expressed in mouse early embryos, confirming the negative off-targets of the siRNAs (Supplementary Fig. S1C).The efficiency of *Ago2* kd was confirmed by q-PCR (Fig. 1B) and immunofluorescence (Fig. 1C). Consistent with previous reports, the development of pre-implantation embryos after *Ago2* deletion was significantly affected from the 4-cell stage (Fig. 1D; *p*-value < 0.05). Then, we performed RNA-seq on the middle 2-cell and early 4-cell embryos with and without *Ago2*. The unsupervised hierarchical clustering (UHC) could separate the *Ago2* kd embryos from the control embryos (Supplementary Fig. S1D, G); meanwhile, the efficiency of *Ago2* kd was further confirmed by RNA-seq data (Supplementary Fig. S1E, H). 365 and 284 genes were increased and decreased in *Ago2*-depleted middle 2-cell embryos, respectively. And in *Ago2*-depleted early 4-cell embryos, 902 upregulated and 1,093 downregulated genes were identified (Supplementary Fig. S1F, I; *p*-value < 0.05). Gene ontology (GO) and Kyoto Encyclopedia of Genes and Genomes (KEGG) pathway analyses were performed on the differentially expressed genes, and the genes were shown to be enriched in cell cycle and transcriptional regulation (Supplementary Fig. S1J, K), verifying the key regulatory role of *Ago2* in developmental progress. We overlapped the genes, whose expressions were increased or stable between MII oocyte and *Ago2*-depleted middle 2-cell embryo, with Cluster I and Cluster III genes, respectively, and found the degradation of 729 and 454 maternal mRNAs in cluster I and cluster III, respectively, was blocked by *Ago2* kd during the MII to 2-cell transition. While, by overlapping the genes, whose expressions were increased or stable between middle 2-cell embryo and *Ago2*-depleted early 4-cell embryo, with Cluster II and Cluster III genes, respectively, we observed the degradation of 290 and 137 maternal mRNAs in cluster II and cluster III, respectively, was inhibited by *Ago2* kd during the transition from middle 2-cell to early 4-cell embryos. The results indicate that the degradation of a large number of maternal mRNAs is AGO2-dependent, especially MII/2 C degradation (Fig. 1E). Additionally, AGO2-dependent MII/2C and 2C/4C degradation was identified in the 2- and 4-cell embryos, respectively (Fig. 1F; Supplementary Table S2), indicating that AGO2-dependent degradation occurs before and along with ZGA. These results show that *Ago2* is involved in MRD in mouse early embryos.

### EndosiRNA is associated with AGO2-dependent MRD

**Fig. 2:**
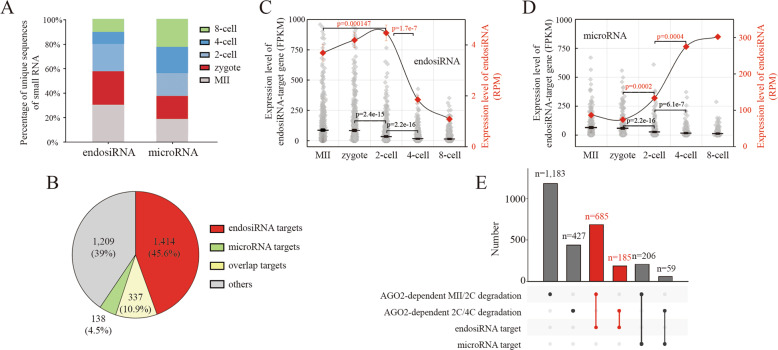
EndosiRNAs are associated with AGO2-dependent MRD. **A** Fraction of unique sequences of endosiRNAs and microRNAs at each stage. The numbers of endosiRNAs sequences were extremely high before the 4-cell stage, while, the numbers of microRNAs sequences were almost equal in each stage. **B** Pie chart showing the fraction of AGO2-related maternal mRNAs targeted by endosiRNAs and microRNAs. Over 50% of the AGO2-related maternal mRNAs were predicted to be targeted by endosiRNAs, while, the number of microRNA target maternal mRNAs is only 475. **C**,**D** Expression patterns of endosiRNAs/microRNAs and their target maternal mRNAs in early mouse embryos. EndosiRNAs were highly expressed at zygote and 2-cell embryo, at that time microRNAs were expressed at low levels and were significantly upregulated until 4-cell stage. **E** Bar plot shows the number of overlaps; the ligature indicates the two groups compared at the bottom.

In the RNAi pathway, AGO2 cooperates with microRNA and endosiRNA, negatively regulating gene expression at the post-transcriptional level^37–44^. Thus, to determine the mechanism of AGO2-dependent MRD, we profiled the unique sequences of microRNAs and endosiRNAs from MII oocytes to 8-cell embryos (GSE83581), and we observed that the numbers of microRNAs sequences were almost equal in each stage. However, the numbers of endosiRNAs sequences were extremely high before the 4-cell stage, consistent with the progress of MRD (Fig. 2A). Then, we screened the small RNAs targeting the maternal mRNAs, and 66,568 endosiRNAs targeting 1,751 maternal mRNAs and 273 microRNAs targeting 475 maternal mRNAs were found (Fig. 2B; Supplementary Table S3). To further confirm the construction of AGO2-endosiRNA network, we analyzed the AGO2 RIP-seq data on mouse embryonic stem (ES) cells, and we found the majority of small RNAs interacting with AGO2 were highly expressed in mouse early embryos (Supplementary Fig. S2A), and about 82% of the 66,568 endosiRNAs targeting maternal mRNAs were directly bounded by AGO2 (Supplementary Fig. S2B), suggesting that endosiRNAs contribute to AGO2-dependent MRD. Moreover, the expression patterns of the endosiRNAs, microRNAs and their targets were analyzed. These data reveal that the targets of both endosiRNAs and microRNAs were significantly downregulated during the transition from the zygote to the 4-cell stage, and correspondingly, the expression peak of endosiRNAs was also observed in the stage (Fig. 2C); however, in contrast, the expression peak of microRNAs was observed at the 4- and 8-cell stages (Fig. 2D). We further explored the role of endosiRNAs and microRNAs on AGO2-dependent degradation and found that 685 and 185 endosiRNA-targeting maternal mRNAs overlapped with AGO2-dependent MII/2C and 2C/4C degradation, respectively; in contrast, only 206 and 59 microRNA-targeting maternal mRNAs overlapped with AGO2-dependent MII/2C and 2C/4C degradation, respectively (Fig. 2E; Supplementary Table S4). The results suggest that endosiRNAs, but not microRNAs, are mainly associated with AGO2-dependent MRD.

### Verification of the role of endosiRNAs in AGO2-dependent MRD

**Fig. 3:**
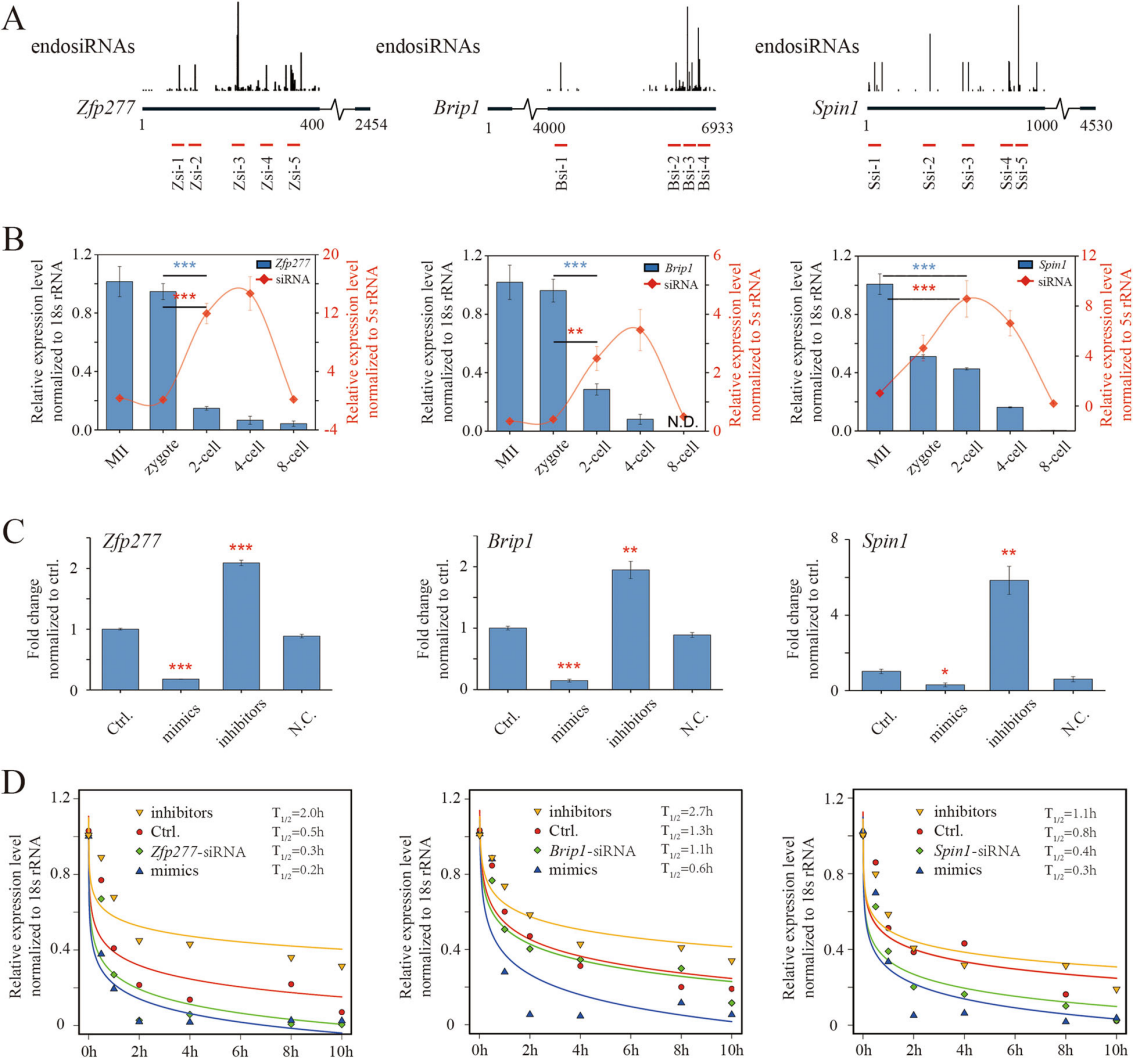
Verification of endosiRNAs in AGO2-dependent MRD. **A** Schematic of *Zfp277*, *Spin1* and *Brip1*. The peak diagram shows the location of endosiRNAs targeting maternal mRNAs, and the red fragment represents the tested endosiRNAs. **B** Q-PCR analysis of the expression patterns of *Zfp277*, *Spin1* and *Brip1* mRNAs and corresponding endosiRNAs in early mouse embryos. Each red dot represents the average expression level of each endosiRNA of individual maternal gene. Three maternal mRNAs degraded prior to the 4-cell stage and were inversely related to the expression pattern of endosiRNA. Error bars indicate the s.e.m. **C** Effect of the corresponding endosiRNAs on the expression levels of *Zfp277*, *Spin1* and *Brip1* measured by q-PCR. Injection of endosiRNA mimics accelerates the degradation of target maternal mRNA, and vice versa. **p < 0.01, Error bars indicate the s.e.m. **D** Degradation curve of *Zfp277*, *Spin1* and *Brip1* obtained by injection of corresponding endosiRNA mimics and inhibitors. Zygotes were treated with actinomycin D (0.6 μg/ml) immediately after microinjection, and were recorded as 0 h. Curve fitting was performed by R package (basicTrendline). The endosiRNAs decreased the expression of the maternal mRNAs by post-transcriptional gene silencing through mRNA degradation. *T*_1/2_ denotes half-life.

To verify the function of endosiRNAs in MRD experimentally, we selected maternal mRNAs of *Zfp277*, *Brip1* and *Spin1*, the degradation of which was AGO2-dependent (Fig. 3A). Their expression patterns and their corresponding endosiRNAs from MII oocytes to 8-cell embryos were examined by q-PCR. The maternal mRNAs were significantly degraded at the 2-cell stage, and the endosiRNAs were present at high levels at this stage (Fig. 3B). And, these endosiRNAs were confirmed to link with AGO2 by AGO2 RIP-seq analysis (Supplementary Fig. S2A). To further confirm the effect of the endosiRNAs on the degradation of the corresponding maternal mRNAs, mimics or inhibitors of the endosiRNAs targeting *Zfp277*, *Brip1*, and *Spin1* mRNAs were injected into zygotes, and the levels of the maternal mRNAs were detected at the 4-cell stage. We found that, compared to the control and negative control (N.C.) groups, the maternal mRNA levels were significantly high when inhibitors were injected and significantly low when mimics were injected, demonstrating that degradation is severely repressed upon the suppression of endosiRNAs and vice versa (Fig. 3C). The results verify the regulatory role of endosiRNAs in AGO2-dependent MRD. Then, we analyzed the stability of *Zfp277*, *Brip1* and *Spin1* mRNAs regulated by the corresponding endosiRNAs. Zygotes were treated with actinomycin D to inhibit transcriptional activity, and the mimics of endosiRNAs were shown to greatly decrease the stability of the corresponding maternal mRNAs; in contrast, the inhibitors could increase the stability (Fig. 3D). These results demonstrate that endosiRNAs facilitate AGO2-dependent MRD at the post-transcriptional level.

### P-bodies participates in endosiRNA-mediated AGO2-dependent MRD

**Fig. 4:**
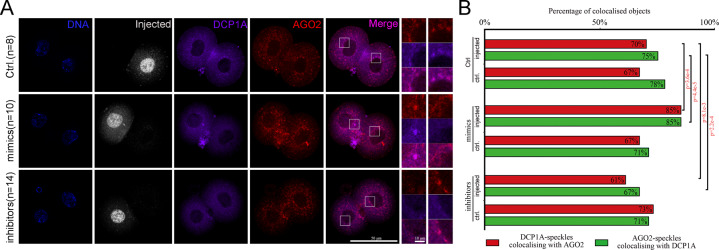
P-bodies participates in AGO2-dependent MRD. A Co-immunostaining of AGO2 with the P-bodies marker DCP1A in 2-cell embryos with injection of endosiRNA mimics or inhibitors mixtures of *Zfp277*, *Spin1* and *Brip1*. Immunofluorescence staining was performed after 1 h of injection. The 5′-Fam labeled negative siRNA was used to mark injected blastomeres (control: *n* = 8; mimics: *n* = 10; inhibitors: *n* = 14). Fluorescence staining was imaged using an inverted confocal microscope. Scale bar, 50 and 10 µm. **B** Quantification of AGO2-positive particle co-staining with DCP1A and DCP1A-positive particle co-staining with AGO2. The colocalization particles of AGO2 and DCP1A increased significantly after injection of mimics, and vice versa.

P-bodies, which are cytoplasmic ribonucleoprotein (RNP) granules, are associated with mRNA decay^45^, and AGO2 is described to interact with P-bodies^46^. Thus, we wanted to check whether P-bodies are related to AGO2-dependent MRD. To this end, we first co-stained AGO2 and DCP1A, the core P-bodies component, in 2-cell embryos. We found a considerable overlap of the staining patterns of AGO2 with DCP1A, identical to the particles residing in perinuclear foci (Fig. 4A), suggesting that the majority of AGO2 was associated with P-bodies. Then, we injected the mixture of the endosiRNA mimics or inhibitors used above into one blastomere of the 2-cell embryos. The 5′-Fam labeled negative siRNA was also injected to mark the injected cells. The localizations of AGO2 and DCP1A were examined after 1 h by immunofluorescence staining. We observed that the colocalization of AGO2 and DCP1A was not markedly changed by the negative siRNA injection in the control group. However, the co-localized particles residing in perinuclear foci of AGO2 and DCP1A were enriched by the injection of endosiRNA mimics, while the percentage of colocalization granules decreased upon injection of endosiRNA inhibitors (Fig. 4A,B). These results reveal that P-bodies may participate in endosiRNA-mediated AGO2-dependent MRD.

### LncRNA complementary to maternal mRNA is involved in the biogenesis of endosiRNAs to facilitate AGO2-dependent MRD

**Fig. 5:**
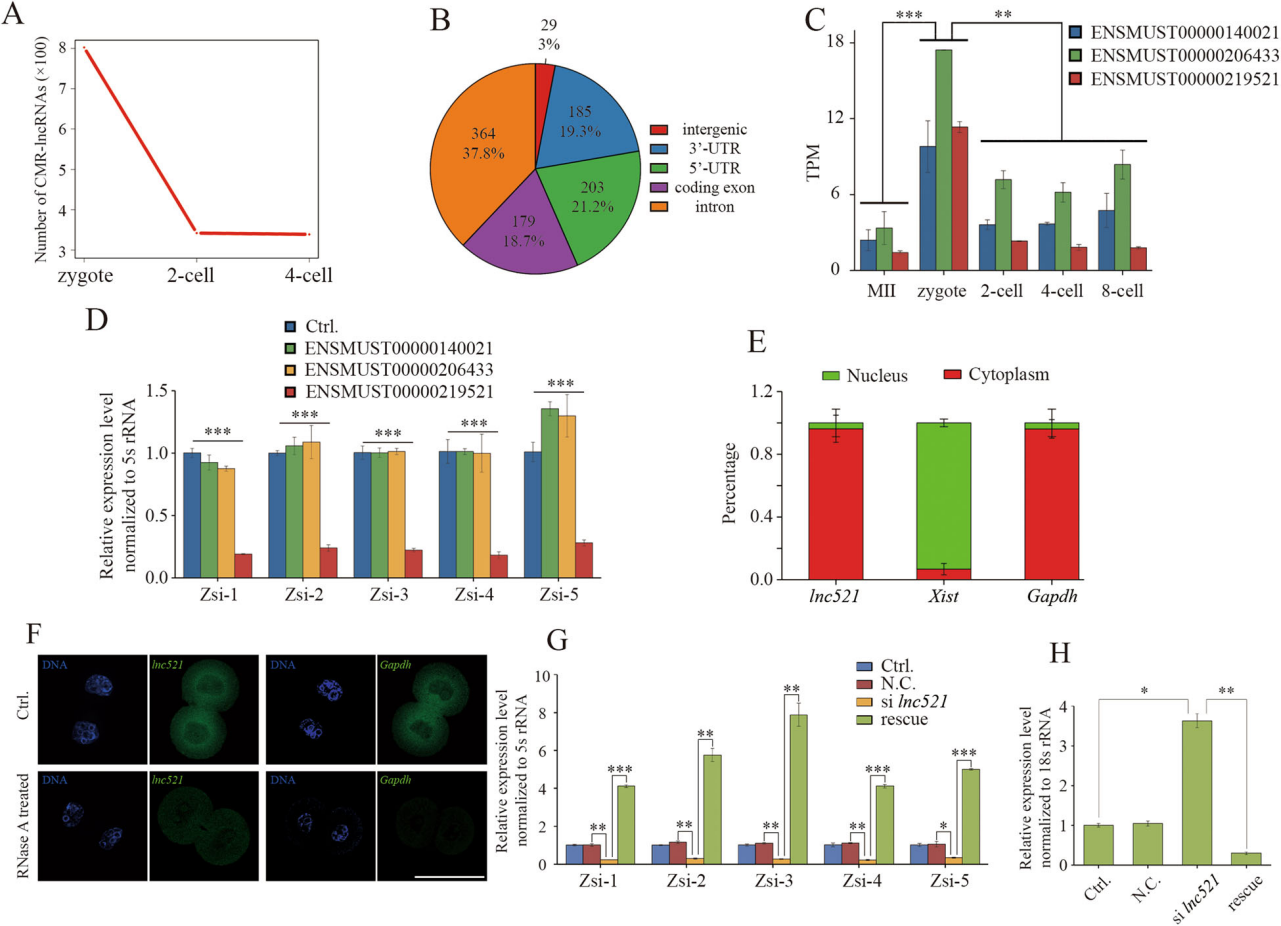
Prediction and verification of CMR-lncRNAs in AGO2-dependent MRD. **A** Number of CMR-lncRNAs predicted in zygotes, 2-cell embryos and 4-cell embryos. The number of lncRNA in zygote is significantly higher than that of 2-cell and 4-cell embryos. **B** Annotation of CMR-lncRNAs in early mouse embryos. CMR-lncRNAs were annotated using all available annotation sources, and a total of 37.8 and 18.7% were found residing in introns and coding exons, respectively. **C** Expression patterns of ENSMUST00000140021, ENSMUST00000206433, and ENSMUST00000219521 according to RNA-seq data in mouse early embryos. **D** Effect of ENSMUST00000140021, ENSMUST00000206433 and ENSMUST00000219521 knockdown on the expression of *Zfp277* endosiRNAs. The results show that deletion of ENSMUST00000219521 (*lnc521*) impaired the biogenesis of endosiRNAs. Error bars indicate the s.e.m. **E** Subcellular localization of *lnc521* by RNA fractionation and q-PCR analysis. Error bars indicate the s.e.m. **F** Representative images of RNA FISH show subcellular localization of *lnc521* in 2-cell embryos (*lnc521*: *n* = 17; *Gapdh*: *n* = 13). RNase A-treated 2-cell embryos were tested as the N.C. (*lnc521*: *n* = 11; *Gapdh*: *n* = 13). Scale bar, 50 µm. **G** Expression levels of *Zfp277* endosiRNAs measured by q-PCR in 2-cell embryos of the control, si *lnc521* injection and si *lnc521* injection with *lnc521* RNA add back. Error bars indicate s.e.m. **H** Expression levels of *Zfp277* measured by q-PCR in 4-cell embryos of control, si *lnc521* injection and si *lnc521* injection with *lnc521* RNA add back (*lnc521* rescue). *p < 0.05; **p < 0.01; ***p < 0.001. Error bars indicate the s.e.m.

Generally, endosiRNAs are processed from dsRNAs formed by the targeted transcripts with the complementary lncRNAs^42,47^. To further demonstrate the AGO2-dependent MRD mediated by endosiRNAs, we screened CMR-lncRNAs in the early embryos. More CMR-lncRNAs, complementary to 403 endosiRNA-targeting maternal mRNAs, were found in zygotes than in 2-cell and 4-cell embryos (Fig. 5A; Supplementary Table S5). CMR-lncRNAs were annotated using all available annotation sources, and a total of 37.8% and 18.7% were found residing in introns and coding exons, revealing that CMR-lncRNAs are likely derived from the intragenic regions of maternal mRNAs to generate endosiRNAs (Fig. 5B). To check the mechanism experimentally, we conducted an in-depth exploration of *Zfp277* and its three predicted CMR-lncRNAs, namely, ENSMUST00000140021, ENSMUST00000206433 and ENSMUST00000219521, the levels of which were particularly high in zygotes (Fig. 5C). To explore the role of CMR-lncRNAs in the biogenesis of endosiRNAs targeting *Zfp277*, we depleted the three CMR-lncRNAs by injecting siRNA into the zygote, and found that endosiRNAs were lacking when an annotated transcript ENSMUST00000219521, termed *lnc521*, was knocked down (Fig. 5D; Supplementary Fig. S2C); the addition of the part of *lnc521* complementary to *Zfp277* mRNA could rescue the deficiency (Fig. 5G). LncRNAs found mainly in the cytoplasm can form dsRNAs^41,42^. Thus, we analyzed the subcellular localization of *lnc521* by q-PCR (Fig. 5E) and RNA-FISH (Fig. 5F) in 2-cell embryos, and the results showed that *lnc521* was mainly located in the cytoplasm. In RNA-FISH, RNase A-treated 2-cell embryos were tested as the N.C. In embryos, the fluorescence signal of *Gapdh* was almost undetectable. However, although the fluorescence intensity was greatly decreased, the fluorescence signal of *lnc521* could still be detected, suggesting that dsRNAs formed by *lnc521* exist in the cytoplasm (Fig. 5F). The results indicate that *lnc521* may participate in the biogenesis of endosiRNAs targeting *Zfp277*. To further investigate the effect of *lnc521* on the degradation of *Zfp277* mRNA, *lnc521* was knocked down, and we found that a deficiency in degradation at the 4-cell stage; this deficiency could also be rescued by addition of *lnc521* (Fig. 5H). Together, these results demonstrate that CMR-lncRNAs may form dsRNA with maternal mRNA and participate in the biogenesis of endosiRNA to facilitate AGO2-dependent MRD.

### AGO2 guided by saRNAs to function in ZGA-dependent MRD

**Fig. 6:**
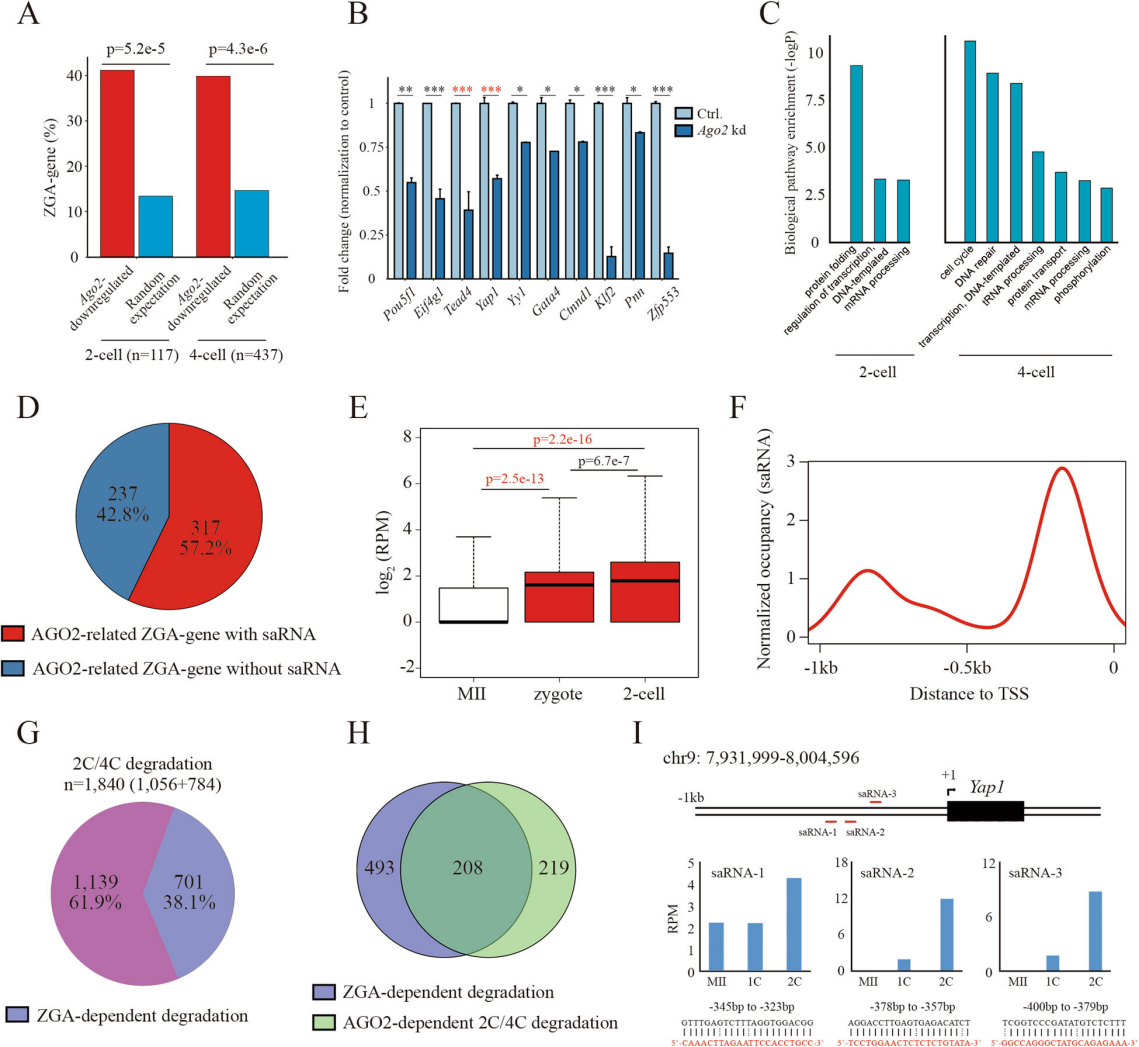
AGO2 guided by saRNAs to function in ZGA-dependent MRD. **A** Bar plot showing the number of ZGA genes embedded within downregulated genes upon *Ago2* deletion and the number expected by chance in 2-cell and 4-cell embryos. **B** Bar plot showing the fold change in ZGA-gene (such as *Yap1*, *Pou5f1* and *Yy1*) expression normalized to the control in 4-cell *Ago2*-deletion embryos. Error bars indicate the s.e.m. **C** GO analysis of the downregulated genes in *Ago2* kd embryos, compared to the control embryo. **D** Pie chart showing the fraction of AGO2-related ZGA genes predicted to be targeted by saRNAs. **E** Box plots showing the expression patterns of the candidate saRNAs in MII oocytes, zygotes and 2-cell embryos. **F** Distribution of saRNAs on the upstream regions of AGO2-related ZGA-gene TSSs. The result shows that the saRNAs mainly recognize the sequences 200-400 bp upstream of the TSSs. **G** Pie chart showing the fraction of 2C/4C degradation depending on ZGA (*n* = 701). **H** Venn diagram showing the overlap between ZGA-dependent degradation and AGO2-dependent 2 C/4 C degradation. **I** Schematic diagram showing the genomic locus of *Yap1* and its saRNA locations. The bar plot shows the expression patterns of the saRNAs. The details of saRNA alignment upstream of the TSS are shown at the bottom.

The above observation showed that the degradation of over half of the maternal mRNAs dependent on AGO2 was mediated by endosiRNAs (Fig. 2E), suggesting that there might be other mechanisms that facilitate AGO2-dependent MRD. AGO2-dependent MRD was observed to occur along with ZGA, and recently, ZGA-dependent MRD was characterized during MZT^16^. In addition, our observation showed that *Ago2*-deletion led to marked developmental decreases after the 2-cell stage (Fig. 1D), indicating that *Ago2* might function in ZGA. Indeed, nearly half of the downregulated genes (*n* = 554) observed upon *Ago2* deletion in 2-cell and 4-cell embryos were ZGA genes identified previously^48^ (Fig. 6A), including *Pou5f1*, *Eif4g1*, *Yap1* and *Yy1* (Fig. 6B), and consistent with previous reports, ZGA-associated biological events were affected (Fig. 6C), suggesting a potential role of *Ago2* in ZGA. *Ago2* guided by saRNAs can target specific promoter regions to stimulate gene expression at the transcriptional level, a phenomenon known as RNAa^24^. Thus, we believe that *Ago2* may cooperate with saRNAs to regulate ZGA. To this end, we screened the candidate saRNAs targeting the upstream sequences within 1.0 kb of AGO2-related ZGA-gene TSSs using small RNA-seq data on mouse MII oocytes and 2-cell and 4-cell embryos. We found that the upstream regions of 317 (out of 554) AGO2-related ZGA genes were predicted to be targeted by saRNAs (Fig. 6D). Compared to those in MII oocytes, the expression patterns of the saRNAs were significantly upregulated in zygotes and 2-cell embryos (Fig. 6E), and consistent with previous reports^24^, the saRNAs mainly recognized the upstream sequences at 200–400 bp of the TSSs (Fig. 6F). These features verified the credibility of the saRNA prediction, and the results suggest that AGO2 may be guided by saRNA to function on ZGA. To assess the ZGA-dependent MRD involved with AGO2, we first identified MRD that depend on ZGA. It has been shown that transiently inhibiting minor ZGA with DRB in zygote (4-20 hpi) severely impaired major ZGA in 2-cell embryos^49^. We found that the expression of a large number of transcripts increased or was stable after the DRB treatment (Supplementary Fig. S2D), and 701 transcripts overlapped with 2C/4C degradation-maternal mRNAs (Fig. 6G), revealing that their degradation is ZGA-dependent. Moreover, 208 of them were also AGO2-dependent 2C/4C degradation maternal mRNAs (Fig. 6H), indicating that *Ago2* may be related to ZGA-dependent MRD. *Yap1* and *Tead4* have been reported to direct ZGA-dependent MRD^16,17^. As expected, the expression of *Yap1* and *Tead4* was significantly downregulated upon *Ago2* deletion (Fig. 6B). We also identified multiple saRNAs with high expression levels in 2-cell embryos targeting the proximal upstream regions of *Yap1* and *Tead4* TSSs (Fig. 6I; Supplementary Fig. S2E). The results suggest that AGO2 may cooperate with saRNAs to activate *Yap1* and *Tead4* and trigger ZGA-dependent MRD.

## Discussion

**Fig. 7:**
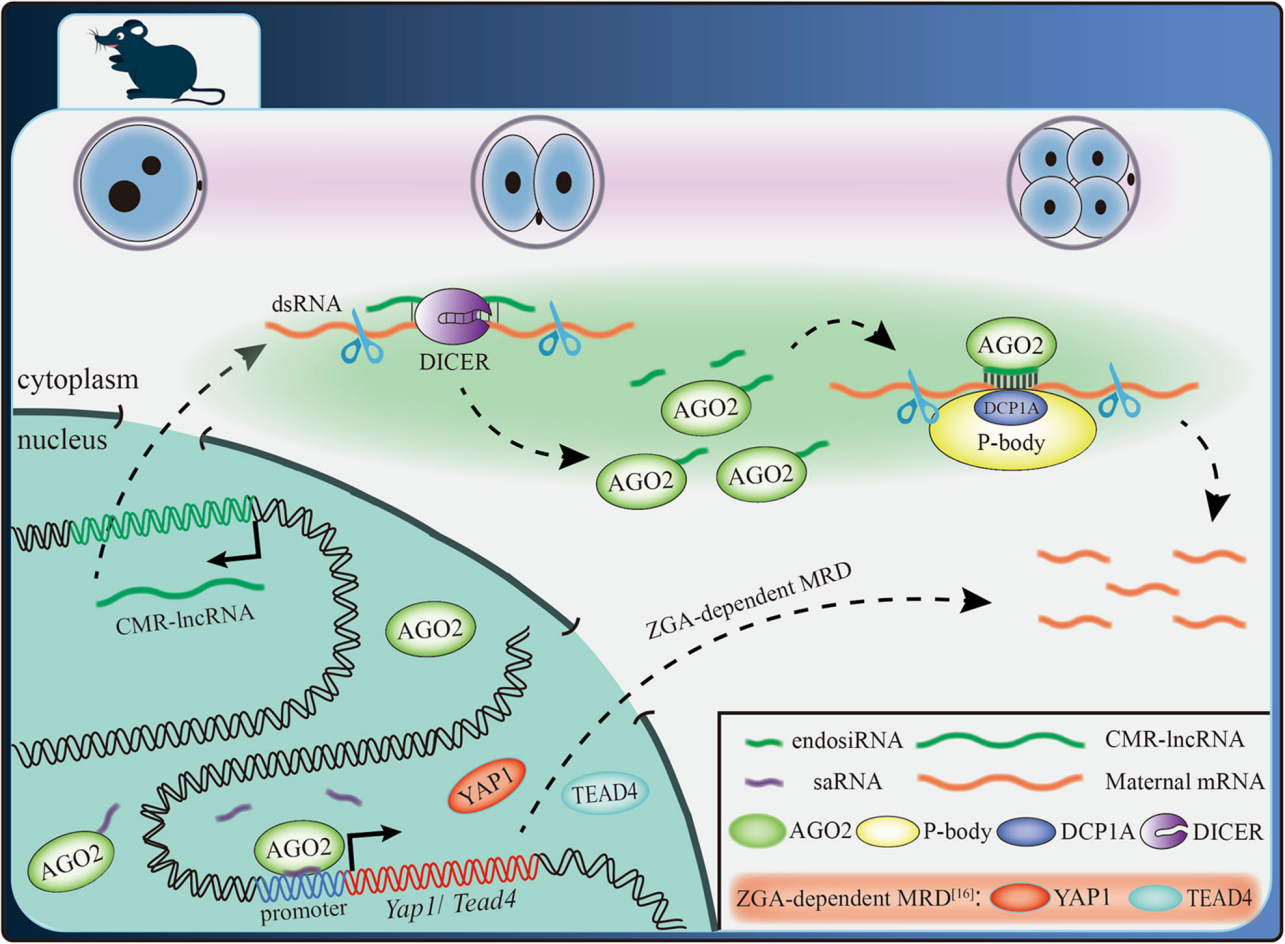
Model for AGO2-dependent MRD. In mouse embryos, AGO2 guided by endosiRNAs, generated from dsRNAs formed by maternal mRNAs with their CMR-lncRNAs, digested with DICER^37^, could target maternal mRNAs and cooperate with P-bodies to promote MRD. In addition, AGO2 may also interact with saRNAs to activate *Yap1* and *Tead4*, and trigger ZGA-dependent MRD^16^.

The transition from maternal to embryonic control of gene expression requires that the amount of accumulated maternal mRNA be greatly reduced. However, maternal mRNAs are inherently stable and remain in oocytes for up to a few weeks before fertilization^8^. Thus, there must be a dramatic change in the stability of the maternal mRNA pool. In this study, we demonstrate that AGO2 guided by endosiRNAs, generated from dsRNAs, formed by maternal mRNAs with their CMR-lncRNAs, digested with DICER^42^, could target maternal mRNAs and cooperate with P-bodies to promote MRD. We also indicate that AGO2 may interact with saRNAs to activate *Yap1* and *Tead4* and trigger ZGA-dependent MRD (Fig. 7). Our findings provide insights into the function of AGO2 in MRD and suggest its role in ZGA.

An important event during MZT is the elimination of a subset of the maternal mRNAs that had accumulated during oogenesis^50^. In both invertebrates and vertebrates, the clearance of maternal mRNAs begins at the onset of oogenesis and continues beyond fertilization^8^. Generally, the clearance of maternal mRNAs is accomplished by two activities: the first is maternally encoded, M-decay, while the second requires zygotic transcription, Z-decay, but it has not been elaborated. AGO2, as a maternal factor, stores in oocyte. In this regard, AGO2-dependent MRD could be related to M-decay. However, AGO2 must be guided by endosiRNAs to bound to the specific maternal mRNAs and perform their degradation, and the production of endosiRNAs may occur in minor ZGA. Furthermore, we also demonstrated that AGO2 functions on ZGA-dependent MRD mediated by saRNAs, which mainly expressed in zygotes and 2-cell embryos. Thus, the AGO2-dependent MRD during the early cleavage seems to be involved in both M-decay and Z-decay, indicating the tight interplay of maternal factor-mediated and ZGA-dependent pathways in spatio-temporal control of MRD. In this study, we showed that *Ago2* regulated the degradation of over half of the maternal mRNAs in mouse early embryos. In the RNAi pathway, AGO2 cooperates with endosiRNAs and microRNAs to mediate post-transcriptional silencing of gene expression^40,42^. However, in mouse early embryos, it has been demonstrated that microRNAs do not contribute to MZT^51–53^. Consistent with this, we found that endosiRNAs, but not microRNAs, are mainly associated with AGO2-dependent MRD.

P-bodies are aggregates of translationally repressed RNPs associated with the translation repression and RNA decay machinery^45,54–56^. We observed the colocalization of AGO2 and P-bodies in perinuclear foci in mouse early embryos, suggesting their common effect on MRD. Furthermore, the enrichment of the co-localized particles residing in perinuclear foci of AGO2 and P-bodies were regulated by endosiRNAs. It has been demonstrated that if the PAZ domain of AGO2, which is necessary for the binding of small RNAs, is destroyed, AGO2 loses its ability to interact with P-bodies^55^. Hence, we indicate that, in addition to bridge AGO2 to maternal mRNAs, endosiRNA is also essential for the cooperation of AGO2 with P-bodies to guarantee the clearance of maternal mRNAs timely.

In agreement with the previous reports^51–53^, we also found endosiRNAs were enriched not only in mouse oocytes, but also in zygotes and 2-cell embryos, suggesting their potential as zygotic sources. We identified a new set of lncRNAs complementary to maternal mRNAs, CMR-lncRNAs, which are involved in the biogenesis of endosiRNAs associated with MRD, and these lncRNAs were mainly found in zygotes, showing the zygotic derivation of endosiRNAs. EndosiRNA from dsRNA formed by basepairing of mRNA and antisense RNA in mouse oocyte and early embryos has been predicted, and suggested to control the pace of clearance of specific maternal mRNAs^57,58^. In the study, we evidenced the endosiRNA-mediated AGO2-dependent MRD pathway. Given that endosiRNAs were produced before ZGA at 2-cell stage, we believe that endosiRNA-mediated AGO2-dependent MRD is independent of the major ZGA. In mice, the products of minor ZGA transcription were characterized between S phase of zygote and G1 phase of 2-cell embryo, and the transcription was relatively promiscuous, low-level, and produced transcripts from thousands of protein-coding genes that were inefficiently spliced and polyadenylated^59^. Consistent with this finding, CMR-lncRNAs were found residing in intragenic regions, making CMR-lncRNAs highly likely to complement maternal mRNAs. Thus, we hypothesize that the CMR-lncRNAs may come from minor ZGA, revealing the regulatory role of minor ZGA in MRD.

Some maternal factors are indicated as driver of ZGA, such as *Brg1*, *Nfya* and *Nelfa*, the deletion of which will result in embryonic developmental arrest at early stages and failure of activation of part of ZGA-genes^18,19,60^. Similarly, the *Ago2* kd led to the developmental defect of majority of embryos after 2-cell stage, and by RNA-seq, we observed that half of the genes downregulated by *Ago2* kd were ZGA-genes. In RNAa, the partnering of AGO2 with saRNA positively regulates gene expression by targeting the promoter region. As expected, we identified multiple saRNAs targeting the upstream of ZGA-genes. Consistent with the previous reports, the target sites were proximal to the TSSs, and the expression of the saRNAs initiated at the zygote and was high in 2-cell embryos, which is consistent with RNAa-induced gene expression being delayed by 24–48 h^24–27^. The results indicate the potential role of *Ago2* in ZGA, which needs to be explored in future studies. ZGA-dependent MRD has been demonstrated in mice, and YAP1-TEAD4 transcription factor-mediated transcription is essential for the degradation^16^. In this study, we found that the degradation of a portion of maternal mRNAs depending on AGO2 occurred along with ZGA, suggesting that *Ago2* may trigger ZGA-dependent MRD as ZGA contributor. In addition, the activation of *Yap1* and *Tead4*, as ZGA-genes, were regulated by *Ago2*, and their saRNAs, located near to the TSSs, were predicted, revealing AGO2 guided by saRNAs may directly activate *Yap1* and *Tead4* to promote ZGA-dependent MRD. This provides a possible mechanism to explain ZGA-dependent MRD mediated by *Ago2*.

In summary, our results demonstrate the novel role of *Ago2* in the degradation of maternal mRNAs during early embryogenesis, and enrich the understanding of RNA metabolism, deepening our understanding of mammalian pre-implantation development, which will help advance the field of reproductive medicine.

## Supplementary information

RNA-seq analysis of 2- and 4-cell Ago2-kd embryos

Knockdown efficiency of lnc521 and prediction of saRNAs

Expression level of maternal gene

Expression level of AGO2-dependent maternal gene

Expression level of small RNA and target maternal gene

Expression level of small RNA and target AGO2-dependent maternal gene

CMR-lncRNA and target maternal gene

## Data Availability

The RNA-seq data in this study were submitted to the NCBI Gene Expression Omnibus (GEO, https://www.ncbi.nlm.nih.gov/geo/) under accession number GSE149785.
