# Supplementary figures and images for: Argonaute 2 is a key regulator of maternal mRNA degradation in mouse early embryos
Source: Cell Death Discov. 2020 Nov 27;6:133. doi: 10.1038/s41420-020-00368-x (PMC7691497; doi:10.1038/s41420-020-00368-x)

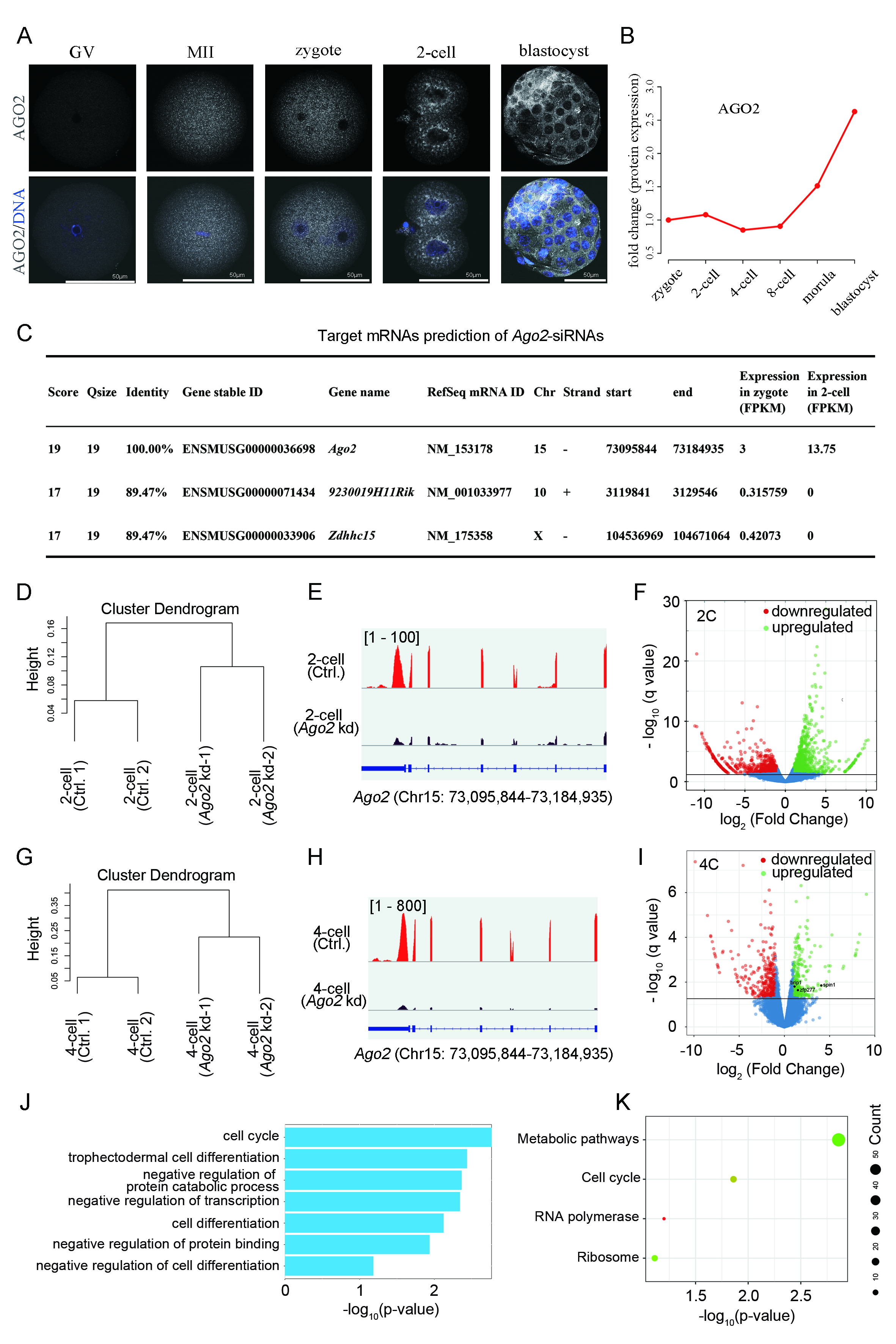

Supplement: Supplementary file 1 — RNA-seq analysis of 2- and 4-cell Ago2-kd embryos [file 41420_2020_368_MOESM1_ESM.tif]

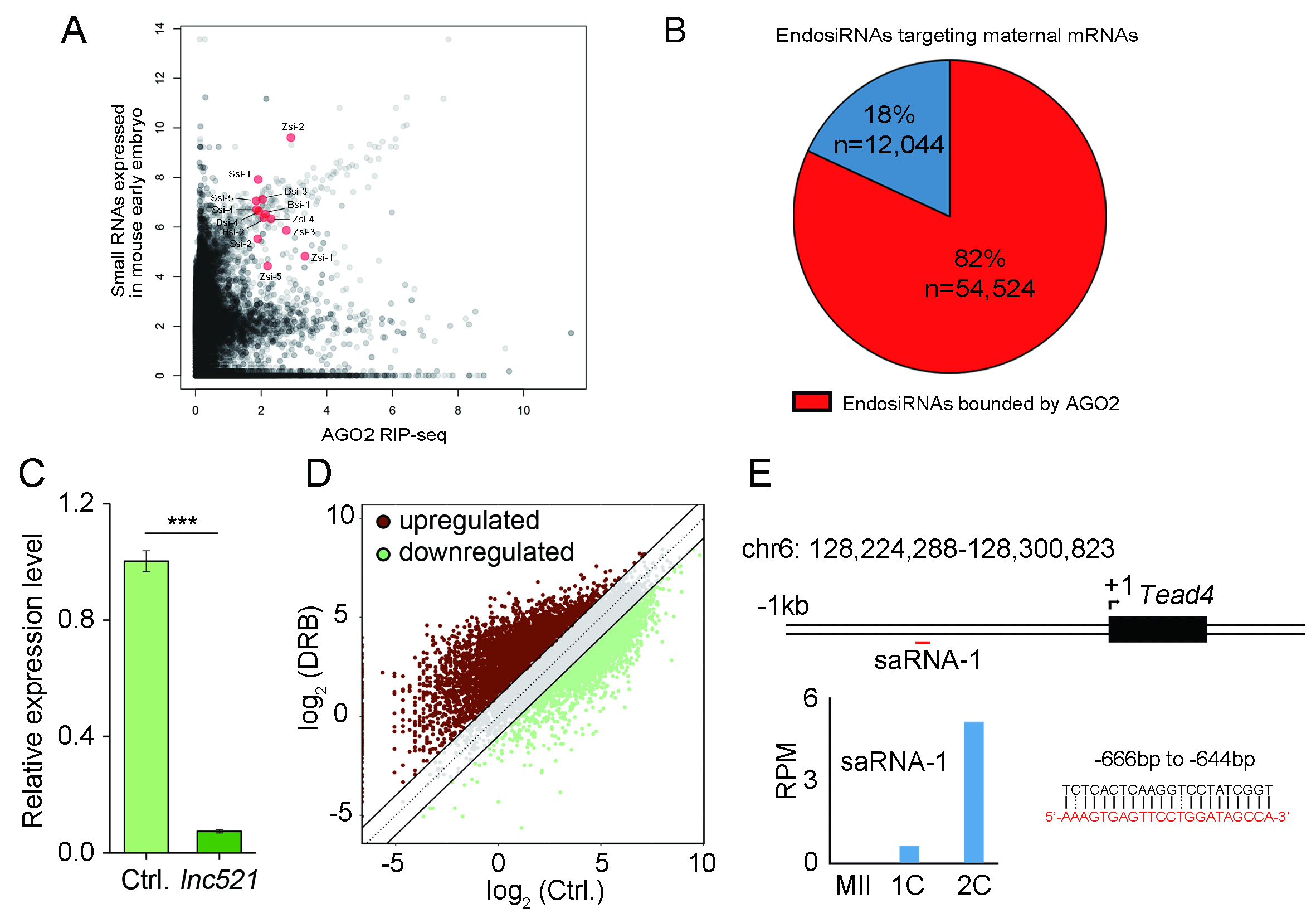

Supplement: Supplementary file 2 — Knockdown efficiency of lnc521 and prediction of saRNAs [file 41420_2020_368_MOESM2_ESM.tif]
